# Comparison of Crystallized Phenol Application and the Karydakis Flap Technique in the Treatment of Sacrococcygeally Localized Pilonidal Sinus Disease

**DOI:** 10.7759/cureus.15030

**Published:** 2021-05-14

**Authors:** Gökhan Akkurt, Hakan Ataş

**Affiliations:** 1 Surgical Oncology, Ankara City Hospital, Ankara, TUR; 2 General Surgery, Ankara City Hospital, Ankara, TUR

**Keywords:** pilonidal sinus treatment, pilonidal sinus disease, crystallized phenol, karydakis flap, sacrococcygeal region

## Abstract

Objective

To evaluate crystallized phenol application and the Karydakis flap procedure in terms of treatment success, postoperative complications, and recurrence in the treatment of pilonidal sinus disease (PSD).

Materials and methods

The study included patients who underwent the Karydakis flap procedure and crystallized phenol application with the diagnosis of PSD in our clinic between June 2016 and January 2019. Age, gender, preoperative body mass index (BMI), sinus number, number of crystallized phenol administrations, postoperative length of hospital stay, postoperative complications, and recurrence rates were compared between the Karydakis and crystallized phenol groups.

Results

Of the 88 patients included in the study, 29 (32.95%) were female, and 59 (67.05%) were male. The median age was 30 (27-33) years, and BMI was 29 (26-32) kg/m^2^. The median sinus number was 3 (2-3). There was no difference between the two groups in terms of age, BMI, sinus number, follow-up duration, gender, recurrence, wound infection, hematoma, wound dehiscence, and preoperative complaints (p > 0.05). A higher rate of drain requirement and more extended hospital stay were observed in the Karydakis group than in the crystallized phenol group, and the difference was statistically significant (p < 0.01 and p < 0.01, respectively).

Conclusion

The crucial advantages of crystallized phenol treatment in PSD are high wound healing rates, outpatient applicability, and no requirement of operating room conditions. Crystallized phenol application also has similar post-application complication rates to the Karydakis flap procedure. The results of our study support that crystallized phenol application is a less invasive alternative treatment method that can be applied before surgical treatment in selected patients.

## Introduction

Pilonidal sinus is a common chronic disease of the sacrococcygeal region, first described in the Boston Medical-Surgical Journal in 1847 by Dr. Andersson [1]. Pilonidal sinus disease (PSD) occurs especially at a young age and is twice as common in men than in women. Studies report the incidence of PSD as 6 per 100,000 in the whole population [2]. Although many factors are associated with the etiology of the disease, the common view is that it is acquired [3,4]. Today, despite the availability of various methods in PSD treatment, none of these methods is clearly superior to the other. The main principles in the treatment of PSD are the simple and easy application of the method to be selected, the short duration of hospital stay, low level of postoperative wound care and pain, low recurrence rates, and quick return to daily activities. In recent years, studies show that flap techniques are superior to primary excision methods in the surgical treatment of PSD [5,6]. Around 35 years ago, Karydakis described the asymmetric closure of PSD. The technique was designed to keep not prevent the suture line remaining above the midline, which is undesirable in primary repair. He also argued that this technique was easily applicable. The suture line remained in the lateral and had several advantages, including early recovery and early return to work and low recurrence rates at 0%-1% [7]. In PSD treatment, crystallized phenol application is a conservative treatment method widely used in many clinics across the world. In addition to being easily applicable, widely accessible, and inexpensive, it is frequently preferred due to low recurrence rates in suitable cases. Phenol is an acidic, mono-substituted aromatic hydrocarbon that has been reported to exhibit antiseptic, anesthetic, and strong sclerosing properties. While it is in crystalline form at room temperature, it can transform into liquid at higher temperatures. The crystalline form is commonly used in the treatment of PSD [5,8]. The aim of the current study was to retrospectively evaluate the data and treatment results of PSD cases treated with the Karydakis flap method and crystallized phenol application.

## Materials and methods

Patients aged 18-50 years who underwent the Karydakis flap procedure or crystallized phenol application with a diagnosis of primary PSD in the Ankara Numune Research and Training Hospital General Surgery Clinic between June 2016 and January 2019 were included in the study. The patients' age, gender, preoperative body mass index (BMI), sinus number, surgical method used, number of crystallized phenol administrations, postoperative length of hospital stay, postoperative complications (wound dehiscence, infection, and hematoma), and postoperative recurrence were obtained by screening the hospital system and contacting the patients by phone. Patients who underwent recurrent operations due to PSD underwent surgery when infected, complicated PSD cases, patients with chronic diseases (diabetes, hypertension, etc.) or cancer history, and those who had received radiotherapy from the pelvic region were excluded from the study. The study's ethics committee approval was obtained from the Clinical Research Ethics Committee of Ankara City Hospital No. 1 (approval number: E1-20-1424). All the patients provided informed consent before the procedure.

Statistical analysis

Data were transferred to the IBM SPSS Statistics v. 23 (IBM Corp., Armonk, NY) for statistical analyses. Non-parametric tests were used since numerical variables were not normally distributed according to the Kolmogorov-Smirnov test. Frequency distribution (number and percentages) was given for categorical variables and descriptive statistics (median and interquartile ranges) for numerical variables. The Mann-Whitney U-test was carried out to examine the differences in numerical variables between the two groups, and the chi-square test was used to investigate the relationship between two categorical variables.

Crystalized phenol application

**Figure 1:**
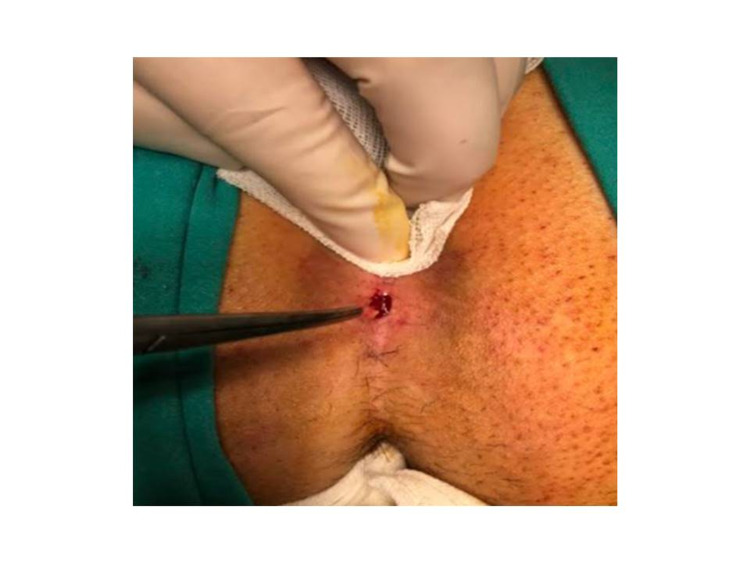
Pit excision and curettage of the sinus.

**Figure 2:**
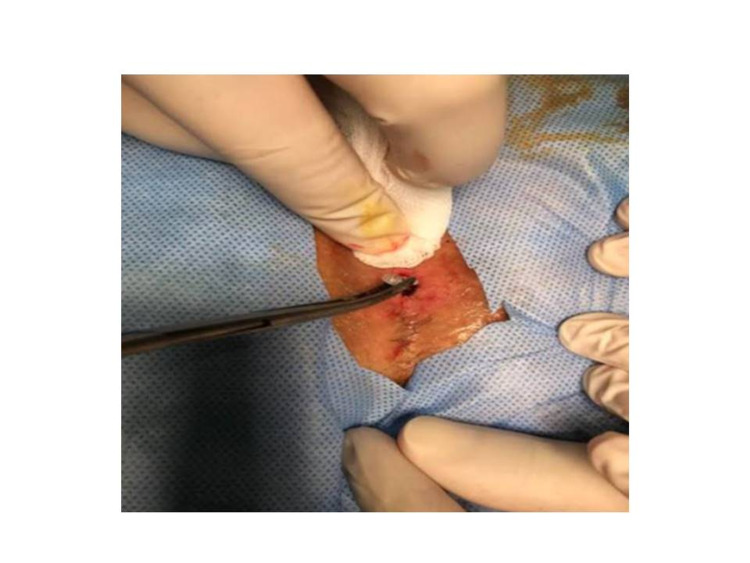
Crystallized phenol application in the treatment of pilonidal sinus disease.

After a sterile site was created and local anesthesia was induced, the sinus tract was cleaned from the sinus openings and curetted. Before the application, the surrounding normal skin tissue was protected with an antibiotic cream (Furacin cream) against pure crystallized phenol's irritating and burning effect. However, non-antibiotic creams can be used for this process. Debris and hair were removed entirely from the sinus (Figure 1). Crystallized phenol was applied into the pilonidal sinus pouch using the Mosquito clamp, and it was observed that the applied crystallized phenol turned into a liquid form. Approximately 3-5 grams of crystallized phenol was applied from each sinus opening, and the procedure was terminated with dressing (Figure 2). The patients were called for control ten days later, at which time the complete closure of the sinus cavity and presence of no discharge was considered as recovery. Crystallized phenol was applied again for a maximum of three sessions at 10-day intervals to the cases in which the cavity was not closed and/or discharge persisted.

Karydakis procedure

**Figure 3:**
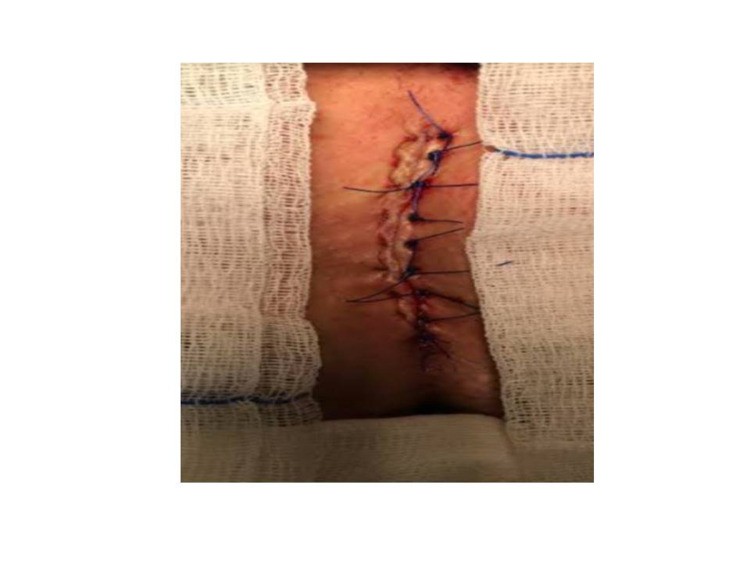
Karydakis flap procedure in the treatment of pilonidal sinus disease.

Following local and/or spinal anesthesia, an elliptical incision with a vertical length of approximately 2 to 5 cm was made above the sinus and 2 cm lateral to the midline. The cyst was carefully excised without damaging the sinus structure and leaving no sinus extension behind. The skin, subcutaneous advancement flap was prepared from the medial side of the incision. The subcutaneous tissue was sutured to the presacral fascia at the base using 2/0 polyglactin (Vicryl) lateralized to the midline at the top. The skin was closed with 3-4/0 polypropylene (Prolene) (Figure 3).

## Results

While there was no statistically significant difference between the groups in terms of age, BMI, sinus number, and follow-up duration (p > 0.05), hospital stay was significantly longer in the Karydakis group compared to the phenol group (p < 0.05). No statistically significant correlation was observed between the type of treatment and gender, recurrence, wound infection, hematoma, wound dehiscence, and preoperative complaints (p > 0.05), but the rate of drain requirement was higher in the Karydakis group than in the phenol group (p < 0.05) (Table 1).

**Table 1:** Descriptive statistics. a,b: indicates the differences between the percentage or median values of the groups (a = highest). k: chi-square test, m: Mann-Whitney U-test, *: p < 0.001.

		Phenol (n = 45)	Karydakis (n = 43)	Test	p
		Median (25-75) n (%)	Median (25-75) n (%)		
Gender	Female	15 (33.3)	14 (32.6)	0.006_k_	0.938
	Male	30 (66.7)	29 (67.4)		
Age		30 (27-33)	30 (27-33)	-0.385_m_	0.700
Body mass index (kg/m^2^)		29 (26-31)	29 (26-32)	0.151_m_	0.880
Sinus number		3 (2-3)	3 (3-3)	1.451_m_	0.147
Recurrence	Absent	43 (95.6)	40 (93)	0.263_k_	0.673
	Present	2 (4.4)	3 (7)		
Wound site infection	Absent	44 (97.8)	41 (95.3)	0.394_k_	0.612
	Present	1 (2.2)	2 (4.7)		
Hematoma	Absent	42 (93.3)	40 (93)	0.003_k_	1.000
	Present	3 (6.7)	3 (7)		
Wound dehiscence	Absent	44 (97.8)	40 (93)	1.146_k_	0.355
	Present	1 (2.2)	3 (7)		
Drain requirement	Absent	45 (100)a	27 (62.8)b	20.465_k_	0.000*
	Present	0 (0)b	16 (37.2)a		
Length of hospital stay (hours)		2 (2-2)b	8 (8-8)a	8.621_m_	0.000*
Follow-up duration (months)		12 (12-13)	12 (12-12)	-0.838_m_	0.402
Preoperative complaints	Absent	7 (15.6)	6 (14)	0.045_k_	0.832
	Present	38 (84.4)	37 (86)		
Number of applications	2	18 (40)	0 (0)	-	-
	3	27 (60)	0 (0)		

In the phenol group, no difference was detected between the genders in terms of length of hospital stay and follow-up duration (p > 0.05). There was also no statistically significant difference in the preoperative complaints and number of applications according to age, BMI, and sinus number (p > 0.05). Furthermore, there was no statistically significant difference between the genders concerning preoperative complaints and the number of applications (p > 0.05). The Spearman correlation analysis revealed that length of hospital stay had a statistically significant negative correlation with age and a statistically significant positive correlation with BMI (p < 0.05) (Table 2).

**Table 2:** Data of the phenol group. r: Spearman's correlation coefficient, k: chi-square test, m: Mann-Whitney U-test, *: p < 0.05.

		Length of hospital stay	Follow-up duration	Preoperative complaints		Number of applications	
				Absent	Present	2	3
		Median (25-75)	Median (25-75)	n (%)	n (%)	n (%)	n (%)
Gender	Female	2 (2-3)	12 (12-12)	1 (14.3)	14 (36.8)	5 (27.8)	10 (37)
	Male	2 (2-2)	12 (12-13)	6 (85.7)	24 (63.2)	13 (72.2)	17 (63)
Test/p		-1.254_m_/0.210	0.303_m_/0.762	1.353_k_/0.395		0.417_k_/0.519	
		r	r	Median (25-75)	Median (25-75)	Median (25-75)	Median (25-75)
Age		-332	114	31 (27-35)	30 (27-33)	30 (27-35)	30 (27-33)
Test/p		0.026*	0.457	-0.251_m_/0.818		-0.035_m_/0.972	
Body mass index		0.322	0.098	26 (24-31)	29 (26-32)	28.5 (25-31)	29 (26-32)
Test/p		0.031*	0.524	1.194_m_/0.246		0.244_m_/0.807	
Sinus number		0.025	-0.137	3 (2-3)	3 (3-3)	3 (3-3)	3 (2-3)
Test/p		0.869	0.370	1.251_m_/0.316		-0.811_m_/0.417	

In the Karydakis group, no statistically significant difference was determined between the genders in terms of length of hospital stay and follow-up duration (p > 0.05), and preoperative complaints did not significantly differ according to age, BMI, and sinus number (p > 0.05). In addition, there was no significant correlation between gender and preoperative complaints and the number of procedures (p > 0.05). Lastly, as a result of the Spearman correlation analysis, no statistically significant relationship was found between length of hospital stay and follow-up duration and age, BMI, and sinus number (p > 0.05) (Table 3).

**Table 3:** Data of the Karydakis group. r: Spearman's correlation coefficient, k: chi-square test, m: Mann-Whitney U-test, *: p < 0.05.

		Length of hospital stay	Follow-up duration	Preoperative complaints	
				Absent	Present
		Median (25-75)	Median (25-75)	n (%)	n (%)
Gender	Female	8 (8-8)	12 (12-12)	0 (0)	14 (37.8)
	Male	8 (8-8)	12 (12-12)	6 (100)	23 (62.2)
Test/p		0.676_m_/0.499	1.103_m_/0.270	3.366_k_/0.155	
		r	r	Median (25-75)	Median (25-75)
Age		0.045	0.139	29.5 (26-31)	30 (27-33)
Test/p		0.775	0.374	0.439_m_/0.669	
Body mass index		0.145	0.083	27 (25-28)	30 (26-32)
Test/p		0.352	0.597	0.985_m_/0.344	
Sinus number		-0.014	-0.161	2.5 (2-3)	3 (3-3)
Test/p		0.931	0.303	1.446_m_/0.277	

## Discussion

Although PSD is primarily seen in the sacrococcygeal region, it can also occur in other parts of our body, including the fingers and toes, navel, and ears [9]. PSD is considered to be an acquired pathology rather than a congenital cyst [10]. The facilitating factors for PSD are as follows: hairy body, an excessive amount of hair shed daily, excessive suction force applied to the hair due to the gluteal cleft being narrow and deep, shed hairs being left in the narrow and deep groove for a long time, long-term skin maceration and moisture leading to ingrown hair, crack, cleft or scar tissue formation in the gluteal cleft, regional trauma due to prolonged sitting, and poor hygiene [11]. Today, many less invasive methods are used together with various surgical techniques in the treatment of PSD. The most critical problem in treatment success is a recurrence, and the search for new methods continues rapidly in many centers. In addition to being simple and easy to apply, the ideal treatment method should not require a long-term hospital stay and post-application care. Also, it should allow rapid return to normal life activities, and most importantly, it should have low recurrence rates [12,13].

In PSD, removing free hair and debris from the sinus and from around the sinus orifice (through shaving and depilation at certain intervals) is the most commonly used non-surgical treatment method. The other frequent procedure is followed by the injection of some chemical agents (80% phenol solution or crystallized phenol, silver nitrate, alcohol, etc.) into the sinus cavity to initiate destruction and promote scar development [14]. On the other hand, surgical methods include Bascom's operation, primary closure after excision, Karydakis technique, sinus excision, marsupialization, and cutaneous graft or flap procedures are the commonly used surgical treatments options [15,16]. Today, minimally invasive methods are widely preferred due to their less invasive nature compared to surgical techniques, applicability under local anesthesia in outpatient conditions, and wide availability and low cost of chemicals used. In the literature, the rate of complete recovery after crystallized phenol application was reported as 86%, and no difference was found between the treatment response and non-response groups in terms of age, gender, and the number of applications [17]. Some authors argue that one-time crystallized phenol application has a lower success rate than surgical procedures but that over 90% can be achieved in treatment through multiple applications [18]. In addition, there is still no consensus concerning the number of crystallized phenol applications required [19].

In our study, we applied three treatment sessions to 60% of the patients and two sessions to 40% in the phenol group. In a study by Kayaalp et al., 30 patients who underwent single-session crystallized phenol application were evaluated, and 93% success was reported after 14 months of follow-up. Doğru et al. reported 95% success in their 24-month follow-up after 107 applications [20]. Another study comparing the excision and Limberg flap method with the phenol application in PSD by Akan et al. determined the length of hospital stay as 1.46 ± 0.61 days in the excision and Limberg flap method. However, they discharged all the patients who were administered crystallized phenol on the same day. In addition, after 26 months of follow-up, there was no statistical difference between the two groups in terms of recurrence [21]. In our study, the mean length of hospital stay was two hours in the phenol group and 8 hours in the Karydakis group. We consider that the most important reason for this is that 36 patients (81.8%) in the Karydakis group were administered spinal anesthesia while only eight were administered local anesthesia. In contrast, local anesthesia was applied to all patients in the phenol group, and the patients were discharged after a short-term follow-up. Furthermore, no drain was used in the phenol group; however, 16 patients (37.2%) in the Karydakis group were required to drain in the operation site before discharge from the hospital. There are also authors arguing that crystallized phenol application can be successfully undertaken not only in primary but also in recurrent cases. In a series of 36 recurrence cases, Aygen et al. reported achieving 92% success with phenol application [19]. But in the present study, we did not include patients with a previous history of abscess due to PSD or recurrence.

The Karydakis procedure, also known as the lateral flap technique, is widely used in PSD due to its easy application and low infection rates, the divergence of the suture line from the midline, and faster wound healing compared to other surgical methods [22]. There are very few studies comparing crystallized phenol application and the Karydakis flap procedure in PSD to the best of our knowledge. In a study by Kurt et al., comparing the crystallized phenol and Karydakis flap methods in PSD, recurrence was not observed in the former, and the recurrence rate was 6.35% in the latter [22]. In our study, recurrences were observed in two (4.4%) patients in the phenol group and three (7%) patients in the Karydakis group. As known wound infection, seroma-hematoma, and wound dehiscence are common problems in PSD cases treated with flaps. Kurt et al. noted the rates of wound infection, seroma-hematoma, and wound dehiscence in patients who underwent the Karydakis procedure as 7.1%, 5.6%, and 3.2%, respectively. But they reported no complication for patients who received crystallized phenol [22]. When we compared our groups in terms of wound infection, postoperative seroma-hematoma, and wound dehiscence, we detected no significant difference. Moreover, side effects such as abscesses and skin burns due to crystallized phenol application mentioned in the literature were not encountered in any patient included in our study [15]. 

The most important limitations of our study include the retrospective design, inclusion of only primary PSD cases, number of sinuses being restricted to three, small number of patients, and short follow-up duration. Also, patients could not be matched exactly in terms of etiological factors (sweating, hair growth, depilation and obesity, etc.) for both groups and that risk matching could not be done in terms of drain use, especially in patients who underwent the Karidakis flap procedure. Since a smaller incision is used and the wound is left open in patients treated with crystallized phenol, there is no need for a drain compared to the Karydakis flap technique. This reduces the significance value of comparing both groups in terms of drain use in our study. Future studies with larger series and more homogenous paired groups will be useful in demonstrating the advantages of crystallized phenol application and Karydakis flap methods used in the treatment of PSD over each other and treatment success rates.

## Conclusions

We consider that crystallized phenol can be used safely in PSD treatment, especially in selected cases. Because it is a more conservative method than other surgical procedures with low recurrence rates and low costs, which also requires only local anesthesia, and allows the patient to return to daily activities earlier.
